# Is perceived social support more important than visual acuity for clinical depression and anxiety in patients with age-related macular degeneration and diabetic retinopathy?

**DOI:** 10.1177/0269215521997991

**Published:** 2021-03-03

**Authors:** Laura Hernández-Moreno, Hugo Senra, Natacha Moreno, António Filipe Macedo

**Affiliations:** 1 Low Vision and Visual Rehabilitation Lab, Department and Center of Physics – Optometry and Vision Science, University of Minho, Braga, Portugal; 2 Centre for Research in Neuropsychology and Cognitive and Behavioural Intervention (CINEICC) – University of Coimbra, Portugal; 3 School of Health and Social Care, University of Essex, Colchester, UK; 4 Hospital Santa Maria Maior E.P.E, Barcelos, Braga, Portugal; 5 Department of Medicine and Optometry, Linnaeus University, Kalmar, Sweden

**Keywords:** Depression, anxiety, social support, age-related eye disease

## Abstract

**Objective:**

To investigate whether visual acuity has the same importance as a factor of depression and anxiety comparing with other psychological variables, particularly perceived social support, in patients diagnosed with age-related eye diseases, with and without low vision.

**Design:**

Observational cross-sectional study.

**Setting:**

Patients attending outpatient appointments at the department of ophthalmology of a general hospital in Portugal.

**Subjects:**

Patients with age-related macular degeneration and patients with diabetic retinopathy attending routine hospital appointments were recruited for this study.

**Measures:**

Anxiety and depression were measured using the hospital anxiety and depression scale and perceived social support using the multidimensional scale of perceived social support. Visual acuity was measured with ETDRS charts.

**Results:**

Of the 71 patients, 53 (75%) were diagnosed with diabetic retinopathy, 37 (52%) were female and age (mean ± SD) was 69 ± 12 years. Acuity in the better seeing eye was 0.41 ± 0.33 logMAR. The mean anxiety score was 4.38 ± 3.82 and depression 4.41 ± 3.39. Clinically significant levels of anxiety were found in 21% (*n* = 15) of the participants and depression in 18%(*n* = 13). The total social support score was 5.29 ± 0.61. Significant multivariate regression models were found for anxiety (*R*^2^ = 0.21, *P* = 0.016) and for depression (*R*^2^ = 0.32, *P* < 0.0001). Social support was independently associated with levels of anxiety and with levels of depression. Gender was independently associated with levels of anxiety.

**Conclusion:**

This study suggests that patients’ perceived social support might be more important than visual acuity as a factor of clinical depression and anxiety in a sample of age-related eye disease patients.

## Introduction

Age-related eye diseases such as age-related macular degeneration, and diabetic retinopathy, commonly occur in people aged 50 years and older,^1^ and can entail permanent disability due to functional limitations caused by vision loss.^2^ Age-related eye diseases are associated with greater risk for mental health problems. According to a recent meta-analysis,^3^ the prevalence of depression in eye disease patients ranges from 5.4% to 57.0% (average 25%). Compared with healthy controls, eye disease patients show an increased prevalence of depression with an odds ratio of 1.59 (95% CI = 1.40–1.81).^3^ Finally, a systematic review of observational studies estimated a prevalence of anxiety in these patients ranging between 9.6% and 30.1%.^4^

Depression and anxiety in patients with age-related eye diseases have been associated with factors such as poor resilience,^5^ use of locus-of-control coping,^6^ social isolation,^6,7^ and poor perceived social support.^6–8^ Some studies have suggested that depression in these patients could be related to reduced visual acuity, that is, depression as a consequence of disability caused by visual impairment.^6,9^ This is consistent with other studies where depression was associated with physical disability.^10,11^ However, the relationship between visual acuity and mental health problems such as depression and anxiety remains unclear, as other studies failed to find any direct relationship between visual acuity and depression.^12,13^

It is, therefore, paramount to clarify underlying causes of depression and anxiety in patients with age-related eye diseases, as this will help clinicians to find effective strategies of managing co-morbid mental health problems associated with vision loss.

In the current study, we want to shed more light on potential factors of anxiety and depression in people diagnosed with age-related macular degeneration or diabetic retinopathy with different levels of visual acuity. Specifically, we want to investigate what is the importance of visual acuity as a factor of anxiety and depression in comparison with other psychosocial variables, particularly social support. We anticipate that patients’ perceived social support is more relevant as a factor of depression and anxiety than visual acuity.

## Methods

The current study is part of an ongoing clinical trial, which has started in March 2017 (registration number: ISRCTN10894889), addressing the cost-effectiveness of a basic vision rehabilitation service in Portugal. The study received approval from the Ethics Committee for Life Sciences and Health of the University of Minho (approval number SECVS 147/2016), and by the Hospital Santa Maria Maior’s ethics committee. The study is registered by the Portuguese data protection authority, with the approval number 7012/2017.

Patients attending outpatient appointments at the department of ophthalmology at Hospital Santa Maria Maior E.P.E (Barcelos, Portugal) were invited to participate in face-to-face interviews. Inclusion criteria: (1) primary diagnosis and cause of vision problem diabetic retinopathy or age-related macular degeneration; (2) 18 years or older and (3) living in the community (not any type of assisted living). The exclusion criteria were: (1) cognitive impairment based on scores of mini-mental state examination, (2) communication problems due to, for example, hearing impairment or inability to speak Portuguese, (3) unable to read due to low level of education. For those accepting to take part, demographic and clinic information data including age, gender and comorbidities was collected. For more information about the complete study design readers are referred to our previous publication.^14^ People were considered to have visual impairment (low vision) if visual acuity in the better seeing eye was less than 0.4 logMAR that is equivalent to a ~15 × 15 mm “H” seen at 4 m (logMAR = Logarithm base 10 of the minimum angle of resolution), and good vision if they meet the standards of physical and mental fitness for driving a motor vehicle in Portugal.^15^ Whilst this varies with the type of vehicle, in our study we established two conditions that should be meet simultaneously: binocular visual acuity better than 0.3 logMAR (equivalent to a ~12 × 12 mm “H” seen at 4 m) and the worst-seeing eye should have 0.7 logMAR (equivalent to a ~29 × 29 mm “H” seen at 4 m) or better acuity.^15^

Symptoms of anxiety and depression were assessed with the Portuguese version of the Hospital Anxiety and Depression Scale.^16^ Hospital Anxiety and Depression Scale is a self- assessment questionnaire, comprising two subscales evaluating levels of depression and levels of anxiety with seven-items each. Each subscale generates scores between 0 and 21, a score above eight indicates the presence of clinically significant levels of anxiety or depression.

Perceived social support was assessed with the Portuguese version of Multidimensional Scale of Perceived Social Support.^17^ This scale has 12 questions that are divided into three subcategories (family, friends, and significant others) with four questions each. The lower the score, the lower the perceived social support.

Distance and near visual acuity were measured with ETDRS charts (Early Treatment Diabetic Retinopathy Study), monocularly at distance and binocularly at near and, in both procedures, a letter by letter scoring was employed.^18,19^ Distance visual acuity was assessed in a dim light room using an internally illuminated cabinet, model 2425E (Precision Vision, IL, USA). Testing distance was adjusted according to the severity of vision loss. ETDRS charts consists of rows of letters, each row comprises five letters and white spaces between letters are equivalent to a letter, each letter corresponds to 0.02 units of acuity and, because of that, letter-by letter scoring can be used.

Data analysis was performed with SPSS (IBM SPSS Statistics for Windows, Version 25.0. Armonk, NY: IBM Corp). Demographic (age, gender, education) and clinical-related variables (diagnosis, comorbidities, visual acuity, depression, anxiety, social support) were summarized for the whole sample. Depression and anxiety variables were square root transformed to reduce right skewness and meet the assumption of normality. *T*-test was used to investigate differences in depression and anxiety scores for gender, education, presence or absence of low vision, comorbidities, and diagnostic type (age-related macular degeneration, or diabetic retinopathy). Pearson’s correlation was used to investigate significant relationships between depression or anxiety and age, distance visual acuity, near visual acuity, and social support scores. Correlation between anxiety or depression and gender was tested using Point-biserial correlation. Assumptions of normality and homogeneity of variance were met for all correlations tested. Multivariate regression analysis was run to identify independent factors of anxiety and depression within our sample. Regression diagnostics were conducted and no outliers were detected. Model’s variance inflation factor values ranged between 1.0 and 8.7, and the tolerance ranged between 0.10 and 0.95, indicating no collinearity among predictors.

## Results

Seventy-one ophthalmological patients (142 eyes) participated in this study, patients’ mean age (±SD) was 68.8 (±11.96) years. Table 1 summarizes sample characteristics and group comparison for depression and anxiety scores. Female patients scored significantly higher on anxiety scale than male patients (*t*(69) = −2.223, *P* = 0.030). Fifteen participants (21%) showed symptoms of clinical depression and thirteen participants (18%) showed symptoms of clinical anxiety. Diabetes (*N* = 59), high blood pressure (*N* = 51), musculoskeletal disorders (*N* = 25) and cardiovascular disease (*N* = 13) were the most frequent comorbid health problems.

**Table 1.** Sample characteristics and univariate analysis.

*N* = 71		Frequency (%)	HADS – Anxiety Mean (SD)*	HADS – Depression Mean (SD)*
Gender	Female	37 (52)	2.1 (.93)**	2.1 (.84)
	Male	34 (48)	1.6 (.89)**	1.7 (.78)
Education	Up to nine years	50 (70)	1.8 (1.08)	1,7 (.89)
	More than nine years	21 (30)	1.9 (.82)	2.1 (.76)
Group	Low vision	38 (54)	1.8 (.98)	2.1 (.79)
	Absence of low vision	33 (46)	1.9 (.87)	1.7 (.85)
Comorbidities	Up to two	30 (42)	1.8 (1.1)	1.7 (.89)
	More than two	41 (58)	1.9 (.81)	2.1 (.76)
Diagnostic	AMD	18 (25)	1.9 (1.0)	1.9 (1.0)
	DR	53 (75)	1.9 (.9)	1.9 (.77)

*Note*. AMD: age-related macular degeneration; DR: diabetic retinopathy.

* T-test compared means of depression and anxiety scores between groups.

** *P* < 0.05.

Best correct distance visual acuity (*r* = 0.24; *P* = 0.044), near visual acuity (*r* = 0.28; *P* = 0.019), and social support (*r* = −0.42; *P*<0.0001) were correlated with depression scores. Social support (*r* = −0.36; *P*<0.0001) and gender (*r* = 0.26, *P* = 0.030) were correlated with anxiety.

Multivariate regression analysis for anxiety (Table 2) led to a final significant model explaining 21% of the variance in patients’ levels of anxiety (*R*^2^ = 0.214, *P* = 0.016), in which gender (*P* = 0.043), and social support (*P* = 0.025) were independently associated with levels of anxiety. Being female and having poorer perceived social support was associated with greater levels of anxiety. The multivariate analysis for depression (Table 3) led to a final significant model, explaining 32% of the variance in patients’ levels of depression (*R*^2^ = 0.32, *P*<0.0001), in which social support (*P* = 0.01) was independently associated with levels of depression. Poorer social support was associated with greater levels of depression. Both regression models were adjusted for demographic factors (age, gender, and years of education), and clinical factors (number of comorbidities).

**Table 2.** Multivariate regression analysis to identify factors of anxiety.

Variable	Unstandardized coefficient (SE)
Age	0.01 (0.01)
Gender	0.44 (0.21)*
Education	–0.35 (0.26)
Distance visual acuity	–0.14 (0.32)
Social Support (MSPSS)	–0.43 (0.19)*
Comorbidities	–0.12 (0.23)

*Note*. *P < 0.05.

**Table 3.** Multivariate regression analysis to identify factors of depression.

Variable	Unstandardized coefficient (SE)
Age	0.002 (0.008)
Gender	0.2 (0.18)
Education	–0.43 (0.22)
Distance visual acuity	0.51 (0.27)
Social Support (MSPSS)	–0.42 (0.16)*
Comorbidities	0.18 (0.18)

*Note*. *P < 0.05.

## Discussion

In this study we investigated factors of anxiety and depression among people with age-related eye diseases with the diagnosis of age-related macular degeneration and diabetic retinopathy. Unlike visual acuity, perceived social support was independently associated with anxiety and depression levels. These findings corroborate our previous hypothesis that perceived social support is more relevant than visual acuity, as a factor of mental health problems in adults with age-related macular degeneration and diabetic retinopathy.

A good perceived social support can have a protective role against mental health problems, as it can enhance psychological resilience to stress.^20^ A deterioration in social functioning, including loneliness, and poor perceived social support, is commonly found in patients with disabling medical conditions such as vision loss,^21,22^ amputation,^23,24^ stroke,^25,26^ and spinal cord injury,^27^ and has been associated with clinical depression and anxiety.^7,8,23–28^ Furthermore, previous studies with these patient groups have highlighted that depression and anxiety can be consequence of patients’ experiences of disability, particularly limitations experienced when performing activities of daily living independently.^9,11,24,26^

Intriguingly, our study findings do not corroborate the previous idea that depression and anxiety are triggered by disability and poor functioning. In our sample, about half of patients still had preserved visual function, that is, they did not have vision-related disability. Despite the heterogeneity of our sample for visual acuity, social support was still a significant factor of depression and anxiety. Furthermore, the prevalence of clinical anxiety and depression in our sample was not much lower than the prevalence found in previous studies where the samples were exclusively composed of patients with disability, such as visual impairment,^3,4^ amputation,^28^ and stroke.^26,29^

Several hypotheses can be raised to explain our findings. Our sample was mainly composed of older adults and we know that depression can be associated with ageing.^30,31^ Previous research suggested that low levels of well-being in older adults with functional impairment are associated with greater risk for further mental health problems such as depression and anxiety.^32^ Low levels of well-being can be a consequence of poor perceived social support, which would explain why in our sample social support was independently associated with depression and anxiety. The great majority of our patients had multi-morbidity, which is associated with high risk for mental health problems including depression and anxiety.^33^ There is some evidence that depression and diabetes are associated and can have a bidirectional relationship.^34,35^ This could explain the rates of depression in our sample as part of it had diabetic retinopathy. Finally, previous research have suggested that depression and anxiety in patients with wet age-related macular degeneration without visual impairment can be triggered by anticipatory anxiety of going blind in the future.^13^

Our study has some limitations such as the size of the sample and the lack of control group (people without any eye diseases). We acknowledge that in the case of our study the sample size may have been a limitation to find this relationship between visual acuity and anxiety and depression. However, our findings raise the debate about the weight of acuity when predicting anxiety and depression in people with age-related eye diseases. This debate is important because, we speculate, most clinicians may believe that drop in acuity is likely to be the main reason why patients’ mental health deteriorates in eye diseases. We believe that the limited sample may have reduced the number of potential factors to be included in the regression model; although, that does not change the main findings of the current study. The lack of a control group limits the extent of our findings because people without age-related macular degeneration and diabetic retinopathy, in particular older patients, are also likely to face problems with mental health and social support.

The main strengths of this study include: (1) a sample composed of patients with different levels of visual acuity which allowed us to examine the potential relationship between visual acuity and mental health outcomes; (2) the use of a robust and widely validated outcome measure for assessing levels of depression and anxiety, the HADS; and (3) the novelty of our findings, since previous literature has mainly suggested that visual acuity and vision impairment are key predictors of anxiety and depression in ophthalmological patients.^6,9^

In summary, our study corroborates the importance that perceived social support has for mental health in patients with disabling medical conditions such as diabetic retinopathy and age-related macular degeneration. Additionally, we raised some hypotheses to explain why, in our sample, visual acuity was not significantly associated with depression and anxiety, which goes against the previous idea that disability is an important factor of depression and anxiety. Future longitudinal studies addressing different disabling medical conditions should clarify which factors underlie the relationship between disability and mental health. This will be key to inform clinical and rehabilitation practice with the aim of preventing long-term mental health problems in these patient groups.

**Clinical messages**

- Symptoms of clinical depression and anxiety in patients with age-related eye diseases might not be a direct consequence of reduced visual acuity;
- Perceived social support plays a key role as factor of depression and anxiety despite heterogeneity of visual acuity in our sample.
